# Molecular diagnosis of opportunistic infections in the central nervous system of HIV-infected adults in Manaus, Amazonas

**DOI:** 10.3389/fmed.2023.1298435

**Published:** 2024-01-09

**Authors:** Sabrina Araújo de Melo, Sérgio Damasceno Pinto, Ewerton da Silva Ferreira, Reinan Brotas, Eveny Perlize Melo Marinho, Valderjane Aprigio da Silva, Rossiclea Lins Monte, Pablo Vinícius Silveira Feitoza, Monique Freire Reis, Taynná V. Rocha Almeida, Luiz Carlos de Lima Ferreira, Michele de Souza Bastos

**Affiliations:** ^1^Universidade Federal do Amazonas, Manaus, Amazonas, Brazil; ^2^Fundação de Medicina Tropical Doutor Heitor Vieira Dourado, Manaus, Amazonas, Brazil; ^3^Universidade Federal do Amazonas, Fundação de Medicina Tropical, Manaus, Brazil; ^4^Departamento de Formação em Emergências em Saúde Pública, Ministério da Saúde, Brasília, Distrito Federal, Brazil

**Keywords:** HIV/AIDS, neurological manifestations, opportunistic infections, cerebrospinal fluid, qPCR (quantitative PCR)

## Abstract

**Background:**

Opportunistic infections in the central nervous system (CNS) of people with HIV/AIDS (PLWHA) remain significant contributors to morbidity and mortality, especially in resource-limited scenarios. Diagnosing these infections can be challenging, as brain imaging is non-specific and expensive. Therefore, molecular analysis of cerebrospinal fluid (CSF) may offer a more accurate and affordable method for diagnosing pathogens.

**Methods:**

We conducted extensive real-time PCR testing (qPCR) on CSF to evaluate etiological agents in PLWHA with neurological manifestations. Primers targeting DNA from specific pathogens, including cytomegalovirus (CMV), herpes simplex virus (HSV), varicella-zoster virus (VZV), Epstein–Barr virus (EBV), John Cunningham virus (JCV), *Toxoplasma gondii*, and human T-lymphotropic virus types 1 and 2 (HTLV-1 and HTLV-2), were used.

**Results:**

Cerebrospinal fluid samples revealed 90 pathogens (36.7%). *Toxoplasma gondii* was the most frequently detected pathogen, found in 22 samples (30.5%). Other pathogens included Cryptococcus sp. (7.7%), EBV (5.3%), CMV, VZV, and JCV (4.0% each).

**Conclusion:**

Despite antiretroviral therapy and medical follow-up, opportunistic central nervous system infections remain frequent in PLWHA. Herpesviruses are commonly detected, but *T. gondii* is the most prevalent opportunistic pathogen in our study population. Therefore, molecular diagnosis is a crucial tool for identifying opportunistic infections, even in patients undergoing treatment.

## Introduction

The human immunodeficiency virus (HIV) was discovered in 1980 and has since remained a significant global public health issue. Globally, approximately 85.6 million people have been infected with HIV since the epidemic’s onset, and currently, almost 39 million people are living with HIV (1). HIV can infect various body parts, including the central nervous system (CNS) (2). Between 40 and 70% of people living with HIV/AIDS (PLWHA) experience neurological manifestations (3). The underlying immunosuppression increases morbidity and mortality, raising the likelihood of infections developing in the central nervous system (4).

Opportunistic infections (OIs) are the primary causes of morbidity and mortality in HIV patients (5). Lack of adherence to antiretroviral therapy and its abandonment further promotes the development of neurological disorders, contributing to a poor prognosis and lower survival rates (6, 7). *Toxoplasma gondii*, cytomegalovirus, *Cryptococcus meningitis*, progressive multifocal leukoencephalopathy (PML), and primary central nervous system lymphoma (PCNSL) are the main agents associated with OIs (8).

Being aware of opportunistic infections with neurological manifestations in PLWHA is crucial. Diagnosing these infections guides appropriate therapy, reduces morbidity and mortality, and prevents long-term sequelae. However, diagnosing these infections can be challenging, as brain imaging, the standard method in most scenarios, is non-specific and expensive. Therefore, analyzing cerebrospinal fluid (CSF) through extensive real-time PCR testing (qPCR) may offer a more accurate and affordable diagnostic method for pathogens. This study aims to describe the main opportunistic pathogens in the central nervous system of PLWHA using qPCR testing in the CSF of patients treated at a reference hospital in Manaus, Amazonas, Brazil.

## Materials and methods

### Study design and population

This observational study occurred at the Fundação de Medicina Tropical Dr. Heitor Vieira Dourado, a tertiary public health institute and a reference center for infectious diseases and cerebrospinal fluid (CSF) analysis. All HIV seropositive patients enrolled in the sexually transmitted infections (STIs) and HIV/AIDS program at FMT-HVD received emergency department treatment.

The study comprised a non-probabilistic convenience sample of 245 patients, aged 18 years or older, with confirmed HIV, regardless of antiretroviral therapy (ART) status. Cerebrospinal fluid samples were collected from FMT-HVD patients with neurological manifestations of the central and/or peripheral nervous system who underwent lumbar puncture between January 2015 and December 2021. These CSF samples were sent to the Bacteriology Laboratory for routine analysis, including total and differential cell counts, determination of protein, glucose, and lactate by spectroscopy, and microbiological tests for bacteria and fungi (smear, culture, latex), as well as molecular diagnosis (qPCR).

Patients were selected based on two criteria: (1) presentation of any neurological symptoms (headache, encephalopathy, convulsive crises, etc.), and (2) performance of lumbar puncture on all patients on the day of admission.

Encephalopathy is defined as altered consciousness persisting for more than 24 h, including lethargy, irritability, or a change in personality or behavior (9).

This study was part of an ongoing nervous system viral infection surveillance program approved by the Ethical Committee of the Fundação de Medicina Tropical Dr. Heitor Vieira Dourado, Manaus, Amazonas, Brazil (CAAE 03929618.8.0000.0005).

### Data collection

We collected information such as sociodemographic data, and clinical, and laboratory results. The categories included signs and symptoms, types of infection, therapeutic regimen, viral load, CD4+/CD8+ T lymphocyte counts, hospitalization period, laboratory tests, and CSF profile. The study also included the diagnosis of *Cryptococcus* sp., *M. tuberculosis* and *syphilis*. All data were obtained via the iDoctor medical record system and internal requests. The information was managed in a database using the software *Research Electronic Data Capture*^©^ (version 12.2.10 Vanderbilt University, 2022).

### Molecular diagnosis

#### Laboratorial diagnosis

Nucleic acid was extracted from a 200 μL CSF sample using the ReliaPrep™ Viral TNA MiniPrep system (Promega, WI, United States). The qPCR reactions were prepared with the GoTaq^®^ Probe 1-Step RT-qPCR System (Promega, WI, United States), using the following protocol: 10 μL of Master Mix, 5.5 μL of water, 1.5 μL of Assay-by-Design (primer and probe set), and 3 μL of DNA to a final volume of 20 μL.

A singleplex PCR was used to amplify specific genes, including herpes simplex type 1 and 2 (HSV-1/2), Epstein–Barr virus (EBV), varicella-zoster virus (VZV), cytomegalovirus (CMV), John Cunningham virus (JCV), BK virus (BKV), *Toxoplasma gondii*, and HTLV-1/2, following the protocols in Table 1 (10–14). After selecting the primer and probe sets, a synthetic positive external control encompassing HSV1, HSV2, CMV, VZV, EBV, *Toxoplasma gondii*, and HTLV1/2 target regions was custom-made by pGBLOCK_1 by IDT DNA Technology (IA, United States).

**Table 1:** Polymerase chain reaction primers and probes.

Pathogen (REF)	Sequence 5′-3′
HSV1-*F* (10)	CGGCCGTGTGACACTATCG
HSV1-R	CTCGTAAAATGGCCCCTCC
HSV1-Probe	CCATACCGACCACACCGACGACC
HSV2-F (10)	CGCTCTCGTAAATGCTTCCCT
HSV2-R	TCTACCCACAACAGACCCACG
HSV2- Probe	CGCGGAGACATCGAGTACCAGATCG
VZV-F (10)	CGGCATGGCCCGTCTAT
VZV-R	TCGCGTGCTGCGGC
VZV-Probe	ATTCAGCAATGGAACACACGACGCC
EBV-F 9 (11)	GGAACCTGGTCATCCTTTGC
EBV-R	ATGGACCGGTTAATCCGATCT
EBV-Probe	AGCGAGCAGTACGAGTGCTGCG
CMV-*F* (11)	CTTAACCACTACAGCAAAGGTACGA
CMV-R	ATGATAGCGGCGTTAGGTGACA
CMV-Probe	TGCCCGAAACGATAGCGTTGCC
JC/BK-*F* (12)	GAAACTGAAGACTCTGGACATGGA
JC/BK-R	GAAACTGAAGACTCTGGACATGGA
JCPyV Probe	AGGATCCCAACACTCTACCACCTAAAAAG
BKPyV Probe	CAAGCACTGAATCAATCACAATGCTC
Toxo-*F* (13)	TCCCCTCTGCTGGCGAAAAGT
Toxo-R	AGCGTTCGTGGTCAACTATCGATTG
Toxo-Probe	TCTGTGCAACTTG GTGTATTCGCAG
HTLV-1-*F* (14)	GAACGCTCTAATGGCATTCTTAAAACC
HTLV-1-R	GTGGTTGATTGTCCATAGGGCTAT
HTLV-1-Probe	ACAAACCCGACCTACCC
HTLV-2-F (14)	CAACCCCACCAGCTCAGG
HTLV-2-R	GGGAAGGTTAGGACAGTCTAGTAGATA
HTLV-2-Probe	TCGAGAGAACCAATGGTATAAT

HSV-1, herpes simplex virus type 1, HSV-2, herpes simplex virus type 2; VZV, varicella zoster virus; EBV, Epstein–Barr virus; CMV, cytomegalovirus; JCV, JC virus; Toxo, *Toxoplasma gondii*; HTLV-1, Human T-lymphotropic virus type 1; HTLV-2, Human T-lymphotropic virus type 2.

The real-time PCR system’s thermocycler conditions were 45°C for 15 min, 95°C for 2 min, followed by 40 cycles at 95°C for 15 s and 60°C for 1 min. Each reaction included CSF samples, a positive external control, a negative control (water), and an internal control consisting of β-actin and RNAse P amplification to validate the presence of nucleic acid.

*Mycobacterium tuberculosis* detection utilized GeneXpert^®^ MTB/RIF (Cepheid, CA, United States). *Cryptococcus* sp. presence in cerebrospinal fluid (CSF) was determined through cryptococcal antigen (CrAg), Indian ink staining, and culture. Neurosyphilis screening involved a Venereal Disease Research Laboratory (VDRL) test on 41 patients, all producing non-reactive results.

### Statistical analysis

A descriptive analysis of the data was conducted. Continuous variables were expressed as median values, and categorical variables were presented as frequencies, both absolute and relative, for the comparison between two or more groups. Data normality was assessed using the D’Agostino test, revealing a non-normal distribution. Consequently, analyses employed the Kruskal-Wallis test, with median comparisons conducted using Dunn’s method, and the Chi-square test to compare variables between HIV patients with confirmed and unconfirmed CSF OIs. The predetermined level of statistical significance was set at *p* < 0.05.

## Results

### Patient characteristics

**Figure 1:**
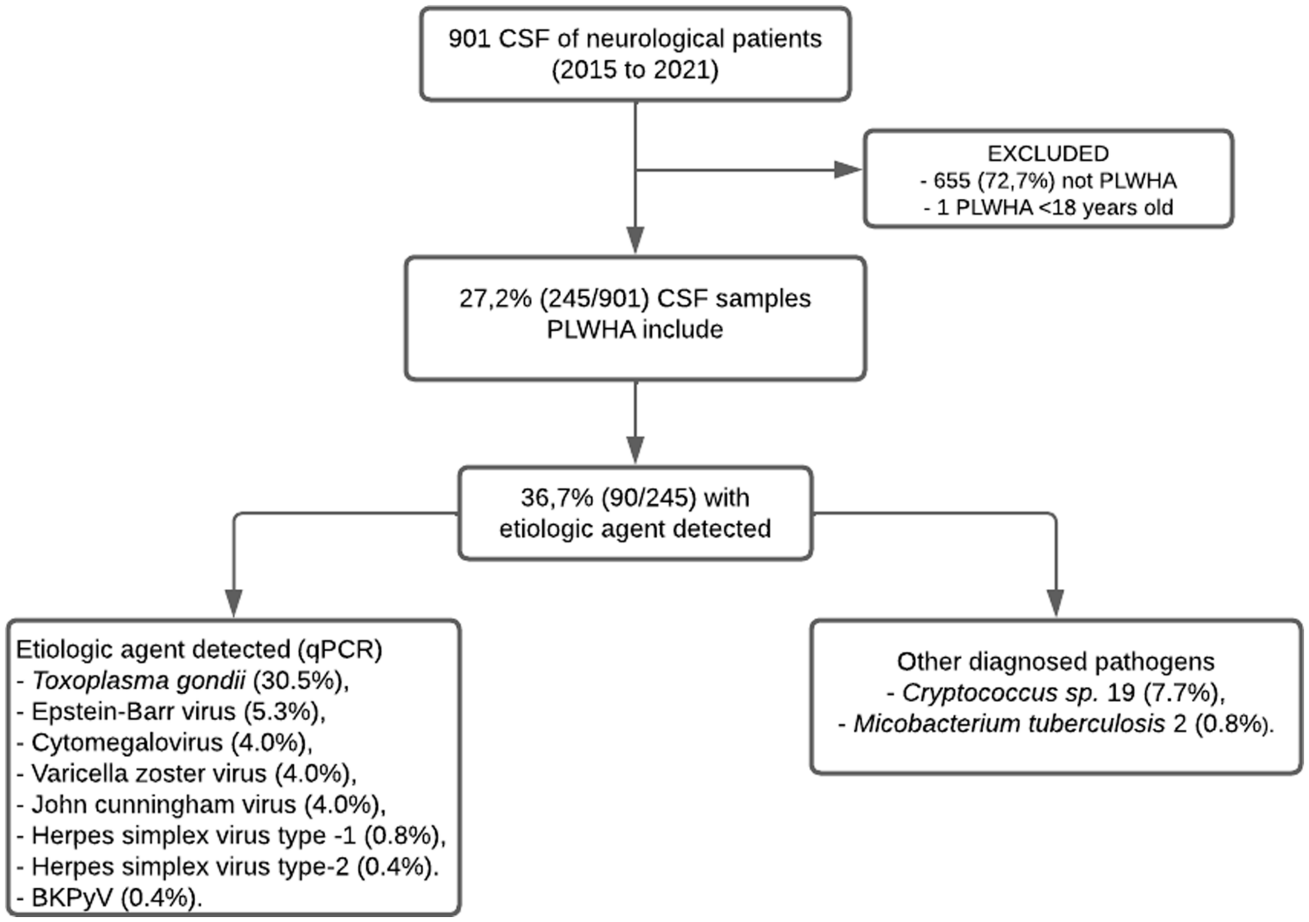
Flowchart of capture, inclusion, and exclusion according to the steps of this research to compose the final sample of PLWHA with suspected CNS infection and neurological manifestations treated at the Fundação de Medicina Tropical Dr. Heitor Vieira Dourado between January 2015 and December 2021.

During the study period, 901 CSF samples from patients with suspected CNS infection were collected. Of these, 656 were excluded for the following reasons: CSF from patients without HIV/AIDS (*n* = 655) and CSF from patients <18 years old (*n* = 1). As a result, we were able to analyze 245 cerebrospinal fluid samples from PLWHA with suspected CNS infection and neurological manifestations (Figure 1).

The cohort, with a median age of 37 (18–72) years, had a 58% male predominant (142/245). Headache was the predominant neurological manifestation in 58.3% (143/245), followed by encephalopathy in 46.5% (114/245), convulsive crises in 23.2% (57/245), and weakness in 20% (49/245) see details in Table 2.

**Table 2:** Neurological manifestations presented by HIV patients with and without detected agents treated at Fundação de Medicina Tropical Dr. Heitor Vieira Dourado from January 2015 to December 2021.

Neurological manifestations*	Total *n* = 245 (%)	HIV with detected agents *n* = 62 (%)	HIV without detected agents *n* = 183 (%)
Headache	143 (58.3)	39 (62.9)	104 (56.8)
Encephalopathy	114 (46.5)	43 (69.3)	71 (38.7)
Convulsive crisis	57 (23.2)	11 (17.7)	46 (25.1)
Weakness	49 (20.0)	17 (27.4)	32 (17.4)
Dysarthria	21 (8.5)	7 (2.8)	14 (5.7)
Aphasia	15 (6.2)	7 (2.8)	8 (3.2)
Photophobia	12 (4.8)	7 (2.8)	5 (2.0)
Memory loss	11 (4.4)	4 (1.6)	7 (2.8)
Neck stiffness	11 (4.4)	7 (2.8)	4 (1.6)
Akathisia	8 (3.2)	3 (1.2)	5 (2.0)

In terms of antiretroviral drugs use, 46.5% of patients reported regular use (114/245), with 25.3% being new to treatment (62/245), 19.8% claiming irregular use (47/245), and 7.7% abandoning treatment (19/245) (Table 3). Considering the HIV infection stage (15), 64% were stage 3 (CD4+ T < 200 cells/mL), 18.5% were stage 2 (CD4+ T 200 to 499 cells/mL), and 6.9% were stage 1 (CD4+ T ≥ 500 cells/mL). Additionally, 10.6% had an unknown stage (no CD4+ T count information). The comparative analysis between HIV patients with confirmed and unconfirmed OIs showed that encephalopathy (*p* ≤ 0.001) and abandoned ART (*p* = 0.042) was significantly higher in the group of patients with detected agent (Table 4).

**Table 3:** Absolute and relative frequency of sociodemographic and clinical characteristics of PLWHA with suspected CNS infection and neurological manifestations treated at the Fundação de Medicina Tropical Dr. Heitor Vieira Dourado between January 2015 and December 2021.

Sociodemographic characteristics	Total (*n* = 245)	(%)
**Sex**		
Male	142	58.0
Female	103	42.0
Neurological manifestations*		
Headache	143	58.3
Encephalopathy	114	46.5
Convulsive crisis	57	23.2
Mental confusion	50	22.4
Weakness	45	20.2
**Use of ART**		
Regular	114	46.5
New to treatment	62	25.3
Irregular	47	19.8
Abandoned ART	19	7.7
Not reported	3	1.2
**Stage of HIV infection/TCD4+**		
Stage 1	17	6.9
Stage 2	45	18.5
Stage 3	157	64.0
Unknown stage	26	10.6
**Plasma viral load**		
Detectable	169	68.9
Undetectable	76	31.0
**Clinical outcome**		
Death	67	27.8
Not death	178	72.6

*The absolute and relative frequency values are subject to variations because the same patient may have had more than one neurological manifestation.

PLWHA, People living with HIV/AIDS.

CNS, Central nervous system.

ART, Antiretroviral therapy.

MMII, Lower members.

**Table 4:** Comparison of demographic characteristics, neurological manifestations, ART utilization, plasma viral load, and clinical outcomes between HIV patients with and without detected agents treated at Fundação de Medicina Tropical Dr. Heitor Vieira Dourado from January 2015 to December 2021.

	Total *n* = 245 (%)	HIV with detected agents *n* = 62 (%)	HIV without detected agents *n* = 183 (%)	*p*-value*
**Demographic feature**				
Sex				
Male	142 (58.0)	32 (51.6)	74 (40.4)	0.160
Female	103 (42.0)	30 (48.4)	109 (59.6)	
Neurological manifestations*				
Headache	143 (58.3)	39 (62.9)	104 (56.8)	0.490
Encephalopathy	114 (46.5)	43 (69.3)	71 (38.7)	**<0.001**
Convulsive crisis	57 (23.2)	11 (17.7)	46 (25.1)	0.300
Weakness	49 (20.0)	17 (27.4)	32 (17.4)	0.132
**Use of ART**				
Regular	114 (46.5)	22 (35.4)	92 (50.2)	0.061
New to treatment	62 (25.3)	16 (25.8)	46 (25.1)	0.948
Irregular	47 (19.1)	14 (22.5)	33 (18.0)	0.548
Abandoned ART	19 (7.7)	9 (14.5)	10 (5.4)	**0.042**
Not reported	3 (1.2)	1 (1.6)	2 (1.0)	–
**Plasma viral load**				
Detectable	169 (69.0)	45 (72.5)	124 (67.7)	0.582
Undetectable	76 (31.0)	17 (27.5)	59 (32.3)	
**Clinical outcome**				
Death	67 (27.3)	18 (29.0)	49 (26.7)	0.857
Not death	178 (72.7)	44 (71.0)	134 (73.3)	

*chi-square test.

CSF, cerebrospinal fluid.

PLWHA, People living with HIV/AIDS.

CNS, Central nervous system.

The bold indicates statistically significant values for encephalopathy (*p* ≤ 0.001) and ART abandonment (*p* = 0.042).

Patient groups with detectable agents were classified by their stage of HIV infection. In all cases, the highest infection incidence occurred in stage 3, identifying patients with AIDS. In the Polyomavirus group, 10 individuals exhibited advanced infection (Table 5).

**Table 5:** Absolute and relative frequency of HIV infection stage classification for detected virus groups.

Pathogen	Individual prevalence /N	(%)
*T. gondii*	22/72	30.5
*Cryptococcus* sp.	19/245	7.7
EBV	13/245	5.3
CMV	10/245	4.0
VZV	10/245	4.0
JCV	10/245	4.0
HSV-1	2/245	0.8
*M. tuberculosis*	2/245	0.8
HSV-2	1/245	0.4
BKV	1/245	0.4
Total	90	36.7

Plasma HIV viral load was detected in 68.9% of patients, with a median of 86,015 copies/ml (range: 14.22–6,290,232). The observed mortality rate in this cohort was 27.8% (67/245). Regarding HIV diagnosis, the median time since infection was 2 years (range: 1–21), characterizing a recent infection in most patients.

### Pathogens detected

PCR testing of the CSF sample revealed pathogens in 90 (36.7%) cases. *Toxoplasma gondii* was the most frequently detected pathogen in 22 (30.5%) samples, followed by *Cryptococcus* sp. in 19 (7.7%), EBV in 13 (5.3%), and CMV, VZV, and JCV in 10 (4.0%) each (Table 6). HSV-1 and *M. tuberculosis* were detected at a rate of 0.8%, and HSV-2 and BKV at 0.4%. Coinfections involved EBV/CMV (2), EBV/VZV (1), VZV/CMV (2), HSV-1/2 (1), and JCV/BKV (1). The molecular diagnosis of *T. gondii* was performed in 72 cases where neurotoxoplasmosis was presumptively diagnosed based on clinical criteria, imaging findings, and therapeutic response.

**Table 6:** Absolute and relative frequency of pathogens detected in 245 CSF samples from PLWHA with suspected CNS infection and neurological manifestations treated at the Fundação de Medicina Tropical Dr. Heitor Vieira Dourado between January 2015 and December 2021.

Infection stage	Herpesvirus *n* = 30 (%)	Polyomavirus *n* = 10 (%)	HIV/CSF *n* = 93 (%)	Coinfection *n* = 7 (%)
Stage 1	1 (3.3)	–	6 (6.4)	–
Stage 2	6 (20)	–	14 (15)	3 (43)
Stage 3	18 (60)	10 (100)	65 (69)	4 (57)
Unknown stage	5 (16.6)	–	8 (8.6)	–

### Cerebrospinal fluid parameters

Biochemical analysis and cytometry of the cerebrospinal fluid samples was performed. The results showed that patients who had herpesviruses had an inflammatory CSF cell profile with high cellularity, with a median of 85.5 cells/mm^3^ (0–3.925) and a significant increase in protein, with a median of 116.1 mg/dL (42.6–1.558) and normal glucose median 61.5 mg/dL (24.6–1.558). Patients with JC *Polyomavirus* infection within the normal CSF profile had protein with a median of 45.8 mg/dL, glucose with a median of 55 mg/dL and cytometry of <5 cells/mm^3^.

CSF parameters of patients with pathogen detection were compared between groups. Protein and cytometry showed statistical significance (*p* ≤ 0.05) in the Herpesvirus group compared to the HIV group (Table 7).

**Table 7:** Comparison of the cerebrospinal fluid profile between the groups of patients who were diagnosed with viral agents in cerebrospinal fluid, including herpesvirus, Poliomavirus, and HIV, and who were treated at the Tropical Medicine Foundation between January 2015 and December 2021.

	Herpesvirus (Group 1) *n* = 30 (M_d_)	Polyomavirus (Group 2) *n* = 10 (M_d_)	HIV no LCR (Group 3) *n* = 93 (M_d_)	*p*		
				1 × 2	1 × 3	2 × 3
Protein (mg/dL)	116.10 (42–1.558)	45.80 (14–201)	850 (80–3.960)	**<0.05**	**<0.05**	ns
Glucose (mg/dL)	61.50 (8–123)	55 (40–68)	53 (8–148)	ns	ns	ns
Lactate (mg/dL)	7.20 (1.5–50)	10.4 (1.6–19.7)	3.10 (0.6–74)	ns	ns	ns
Cytometry (cell/mm^3^)	104 (0–3.925)	0 (0–5)	17 (0–874)	ns	**<0.05**	ns

The bold indicates statistically significant values for protein and cytometry (*p* ≤ 0.05).

## Discussion

The introduction of ART significantly reduced opportunistic CNS infections. Nevertheless, diagnosing and treating these infections remains challenging, posing a substantial threat to morbidity and mortality, particularly among individuals with untreated or undiagnosed HIV. This retrospective study examines the predominant pathogens in the cerebrospinal fluid of HIV/AIDS patients at a reference hospital in Manaus, Amazonas.

For people with HIV, their AIDS status is closely linked to opportunistic infections that can induce neurological diseases. Despite the widespread distribution of ART in Brazil, there persists a notable prevalence of opportunistic neurological diseases (16). Our study revealed that 36.7% of cerebrospinal fluid samples from HIV-positive patients with neurological manifestations contained opportunistic pathogens. The Herpesviridae family, at 12.2%, was the most prevalent, followed by Toxoplasma gondii at 8.9%, and Cryptococcus sp. at 7.7%. JCV accounted for 4%, and *Mycobacterium tuberculosis* comprised 0.8%. Our findings underscore the importance of molecular diagnosis for identifying opportunistic agents causing central nervous system infections in PLWHA, regardless of ART use.

Herpesviruses can cause CNS diseases during primary infection or reactivation, with a high prevalence in high-risk populations, especially PLWHA (17, 18). These viruses are strongly associated with serious infections, and their reactivation is almost always linked to negative outcomes. Therefore, an early and accurate diagnosis is essential to identify this viral group (18, 19). In our study, herpesvirus accounted for 12.2% of opportunistic CNS infections in PLWHA, which is lower than reported in the literature. Yang et al. (20) found herpesviruses responsible for 26.6% of neurological disease cases. Gaeta et al. (21) reported herpesvirus DNA detection in 33.5% of CSF samples they analyzed. The lower percentage in our study could result from prior antiretroviral drug use, potentially eliminating the virus and rendering nucleic acid undetectable.

Epstein–Barr virus has a global distribution, with estimates suggesting approximately 90% prevalence in the adult population. Complications from EBV infection in the CNS are rare, ranging from 0.5 to 7.5% as the first or only neurological manifestation (22). Literature indicates that detecting EBV DNA in CSF may be linked to active virus replication in B lymphocytes and an elevated risk of death (20). However, some authors caution that detecting EBV in CSF may be uncertain, as it is unclear whether the virus presence signifies active replication or latent virus in B cells transported during immune surveillance and inflammatory processes (23).

In our study, EBV DNA was detected in 5.3% (13/245) of the samples, revealing an inflamed cerebrospinal fluid profile with high protein (173 mg/dL) and cellularity (122 cells/mm^3^) indices. Wang et al. (23) reported significantly higher CSF protein concentration in patients with detected EBV compared to those without. Opintan et al. (24) found a 45.2% (38/84) EBV prevalence in their tested samples but lacked CSF profile data for these patients. Conversely, in a Malawi study by Benjamin et al. (25), a high EBV incidence (36%) was observed in HIV-positive patients, even with a normal CSF profile. Additionally, we identified EBV in three coinfections, two with CMV and one with VZV, suggesting potential active replication or reactivation due to the presence of opportunistic agents (22).

Reactivation of VZV in the CNS is associated with serious complications, typically seen in acute meningoencephalitis. In PLWHA, this reactivation is more prevalent than in immunocompetent patients, often linked to low CD4+ T-cell counts and the appearance of skin lesions preceding neurological involvement (26). In our study, VZV DNA was detected in 4% of samples. A Zambia study analyzing samples from 331 HIV patients found a VZV detection prevalence of 3.9% (13/331) (11).

CMV is widespread globally, causing mild symptoms in immunocompetent individuals. However, in immunocompromised patients, especially PLWHA, it can lead to severe illness and death (27). Despite ART’s introduction reducing CMV-related neurological complications, its presence persists. Factors contributing to this include non-adherence to ART, antiretroviral resistance, and late HIV diagnosis (28). Neurological complications are more frequent in patients with low CD4+ T-cell counts (<50 cells/mm^3^), often coinciding with symptoms elsewhere in the body.

In our study, CMV DNA was detected in 4% of samples (10/245). In these patients, the median CD4+ T-cell count was 84 cells/mm^3^ (6–461), and 80% had a detectable plasma viral load with a median of 60,962 copies/mL (176–1,186,064). Six patients (40%) in this group succumbed. A study in São Paulo’s reference center reported a 2.4% (6/248) prevalence of cytomegalovirus detection, defining them as CMV encephalitis patients with CD4+ T-cell counts <50 cells/mm^3^ (29). Another prospective observational cohort study in a Brazilian tertiary health center, involving 105 PLWHA, identified CMV as the third most frequent pathogen (12%), responsible for encephalitis, polyradiculitis, and/or retinitis (16).

PML is a demyelinating disease resulting from JC virus reactivation in immunocompromised patients, especially PLWHA (30). Characterized by focal neurological deficits like hemiparesis, gait disturbance, visual disorders, and mental issues (31), HIV-associated neurological diseases affect up to 5% of untreated HIV patients, classified as an AIDS-defining condition (32). Although PML incidence has reduced among PLWHA using ART, it remains the fourth most common neurological complication in Brazil (33, 34).

In our study, JC virus detection frequency was 4% (10/245). Similar results were found by Vidal et al. (35) in a São Paulo study, reporting 6% (12/219) PML cases among patients with neurological disease. Another study in Goiás with 45 suspected PML HIV-positive patients confirmed JC virus presence in the CSF of 11.1%. The mean CD4+ T-cell count was 54 cells/mm^3^, and median HIV viral load was 91,984 copies/mL. Two patients exhibited abnormal CSF analysis, with pleocytosis (20–288 cells/mm^3^) and one case with high protein (32).

Our study showed a 50% mortality rate, predominantly in females, with 80% detecting JC virus. These patients had severe immunosuppression, with a median CD4+ T-cell count of 120 cells/mm^3^ (range: 31–378) and high HIV plasma viral load (median: 4,551 copies/mL, range: 8–542.71). Despite normal CSF profiles (median protein: 45 mg/dL, median glucose: 55 mg/dL, median cellularity: 0 cells/mm^3^), emphasizing the need for molecular differential diagnosis in suspected PML cases, even with a normal CSF profile. Furthermore, irregular ART use was reported in 50% of cases, likely contributing to the high mortality rate since PML lacks specific treatment, and clinical improvement correlates with regular ART use (36).

The most common causes of sporadic encephalitis in immunocompetent adults globally are herpes simplex type 1 and 2 viruses, although they seldom cause encephalitis in HIV patients (37). In our study, HSV-1 was detected in only two patients (0.8%), and HSV-2 was found in a coinfection with HSV-1. Similar findings were noted by Benjamin et al. (25), who identified two cases of HSV-1 and no cases of HSV-2 in suspected viral meningitis cases. Literature suggests HSV-1’s preference for the frontal and temporal lobes, leading to likely emotional and behavioral neurological manifestations. In contrast, HSV-2 is typically associated with meningitis (17). Our study observed manifestations of herpetic encephalitis, including decreased levels of consciousness, mental confusion, and neck stiffness, aligning with existing literature data.

Neurotoxoplasmosis is the most common opportunistic infection causing brain lesions in people with advanced immunosuppression lacking prophylactic treatment (38). It results from *Toxoplasma gondii* reactivation latent in tissue cysts, occurring in 3–40% of PLWHA (38, 39). In our study, only 72 patients received a clinical neurotoxoplasmosis diagnosis based on clinical criteria, imaging findings, and therapeutic response. *T. gondii* DNA was confirmed in 30.5% (22/72) of CSF samples, being the most detected pathogen. Similarly, a Ghana study reported 25% (21/84) of HIV-positive patients testing positive for *T. gondii* through molecular diagnosis (24). Telles et al. (16) found *T. gondii* as the most commonly detected pathogen in CSF samples (36%) in a São Paulo study. Frequent neurological manifestations in patients with confirmed *T. gondii* DNA included headache (66.7%), limb paresis (28.5%), lowered levels of consciousness, aphasia (23.8%), and seizures (19%). In our study, the median CD4+ T-cell count was 78 cells/mm^3^ (21–1,128), and HIV plasma viral load was detected in 89% with a median of 130,070 copies/mL (43–3,671,298). Studies reported an increased frequency of neurotoxoplasmosis in HIV patients with a CD4+ T-cell count below 100 cells/mm^3^ (40, 41).

In our study, we observed that patients with encephalopathy and those abandoning ART were predisposed to detecting an opportunistic agent concomitant with HIV. Comparative analysis on patients with confirmed and unconfirmed OIs revealed significantly higher rates of encephalopathy (*p* = 0.001) and ART dropout (*p* = 0.042) in the group with detected agents. These findings align with published literature, highlighting that despite the substantial reduction in OI incidence and prevalence in the central nervous system due to available antiretroviral therapy (ART), patients remain susceptible to neurological manifestations, particularly upon abandoning of antiretroviral treatment (31).

In this study, the overall mortality rate was 27.8%, lower than the 35.3% reported by Siddiq et al. (10) in hospitalized patients. Importantly, our study is cross-sectional, and we did not follow patients long-term. Therefore, mortality is not only related to CNS infection, especially in the group of individuals with no identified etiologic agent. We emphasize that patients with HIV have overlapping infections in addition to the CNS and may be responsible for mortality in both groups evaluated. In this study our aim was to describe the main opportunistic pathogens in the central nervous system of PLWHA.

The prevalence of opportunistic neurological infections in PLWHA varies based on immune status, antiretroviral treatment adherence, and geographic factors. Signs and symptoms may lack specificity, complicating clinical or laboratory diagnosis. Thus, understanding the agents responsible for infections in PLWHA is essential.

Our study had limitations due to its retrospective nature, leading to the absence of certain clinical and laboratory information. Lack of follow-up prevented an accurate assessment of the clinical history. Despite an extensive diagnostic workup, limitations in qPCR diagnostic capabilities restricted the identification of specific pathogens in this population. Thus, additional pathogens could be associated with opportunistic neurological infections in this population.

In conclusion, this study underscores the significance of knowledge about opportunistic agents in central nervous system infections among PLWHA. Despite ART usage, the prevalence of opportunistic neurological diseases was high at 36.8%, with *Toxoplasma gondii* and herpesviruses identified as the most common causes. Additionally, 64% of these patients had advanced HIV infection. Diagnosing these infections may pose challenges due to non-specific and expensive brain imaging. Therefore, employing molecular tests on CSF samples may offer a more accurate and affordable diagnostic method for pathogens. Utilizing sensitive and fast techniques for laboratory surveillance is crucial to identify agents causing neurological disease, facilitating improved clinical decision-making and the selection of appropriate therapy for these patients.

## Data availability statement

The raw data supporting the conclusions of this article will be made available by the authors, without undue reservation.

## Ethics statement

The studies involving humans were approved by Ethical Committee of the Fundação de Medicina Tropical Dr. Heitor Vieira Dourado, Manaus, Amazonas, Brazil (CAAE 03929618.8.0000.0005). The studies were conducted in accordance with the local legislation and institutional requirements. The human samples used in this study were acquired from primarily isolated as part of your previous study for which ethical approval was obtained. Written informed consent for participation was not required from the participants or the participants’ legal guardians/next of kin in accordance with the national legislation and institutional requirements.

## Author contributions

SM: Methodology, Writing – original draft. SP: Methodology, Writing – original draft. EF: Writing – review & editing. RB: Writing – review & editing. EM: Writing – review & editing. VS: Writing – review & editing. RM: Methodology, Writing – review & editing. MR: Resources, Writing – review & editing. TA: Methodology, Writing – review & editing. LF: Funding acquisition, Resources, Writing – review & editing. MB: Methodology, Supervision, Writing – review & editing. PF: Data curation, Methodology, Writing – review & editing.
